# Insights into values and emotional wellbeing of medical students in the United Arab Emirates: a cross-sectional study

**DOI:** 10.3389/fpsyg.2024.1428115

**Published:** 2024-08-29

**Authors:** Fatma Mustafa Alhashimi, Sara Salim, Warda Siddiqi, Lakshmanan Jeyaseelan, Nusrat Khan, Meshal A. Sultan

**Affiliations:** College of Medicine, Mohammed Bin Rashid University of Medicine and Health Sciences, Dubai, United Arab Emirates

**Keywords:** values, emotions, wellbeing, medical students, education

## Abstract

**Objective:**

To describe the difference in values among medical students from a first-year student’ and final year student’ perspective. In addition, it is designed to report associations and trends between personal values and overall emotional states.

**Methods:**

This is an analytical cross-sectional study that involved disseminating an online survey via email to first and final year students at the College of Medicine in Mohammed Bin Rashid University (MBRU) in Dubai, United Arab Emirates in December of 2023. The survey encompassed queries on demographics, the Life Values Inventory (LVI) and the Positive and Negative Affect Schedule (PANAS).

**Results:**

The survey was completed by 84 students. About half of the participants were final year medical students (45/84; 53.6%) with the majority being females (70/84; 83.3%). Positive emotions were positively correlated to various life values, including belonging, scientific understanding, responsibility, and achievement (*p* < 0.05). When comparing academic years, the scores of the life value of Achievement showed a significant correlation (*p* = 0.04), with first-year students’ mean (SD) of 12 (2) out of 15 compared to 11 (3) out of 15 for final-year students. Positive Emotions also exhibited a significant correlation (*p* = 0.006), with first-year students’ mean (SD) 40 (5) out of 50 compared to 36 (7) out of 50 for final-year students.

**Conclusion:**

This study adds to medical education research by exploring values and emotions, shedding light on factors shaping students’ professional identities. Understanding these dynamics can aid in supporting future healthcare providers and by extension the patients for whom they care.

## 1 Introduction

Values are broad motivational constructs that express what is important to individuals, influencing how they perceive their environment and their self-identity (Bilsky and Schwartz, 1994). They provide internalized standards for judgment and are a primary driving force in motivation, impacting self-regulation and evaluation of rewards (Hitlin, 2003). For medical students, values not only shape professional decisions but also affect emotional well-being (Twenge, 2009; Eley et al., 2016).

The medical field has experienced significant changes, including technological advancements, shifts towards patient-centered care, and evolving ethical considerations, prompting students to reassess their values (Hojat et al., 1998). Limited research exists on the correlation between medical professionals’ values and their decision-making, focusing more on broad ethical dilemmas than practical scenarios encountered in clinical practice (Helkama et al., 2003).

Current research connects values with competencies like problem-solving in nursing students, ethical decision-making among medical and dental students, and moral maturation of medical students (McCabe et al., 1992). Educators suggest that understanding personal values can mitigate biases and enhance patient-focused decisions (Duggan et al., 2006; Epstein, 1999). Medical education now emphasizes a holistic approach, fostering values such as empathy, integrity, and resilience.

This study examines differences in values between first-year to final-year medical students and their correlation with emotional states. Previous studies have looked at factors like sleep quality, physical activity, and coping strategies (Meer et al., 2022; Alhusami et al., 2024; Boushehri et al., 2024), but research on the relationship between values and emotions is limited. Understanding these trends is crucial, especially in the United Arab Emirates (UAE), where such studies are scarce, addressing a vital gap in our knowledge of the dynamic interplay between values and emotions in medical students.

### 1.1 Aim

The overall aim of this study is to describe the difference in values among medical students from a first-year students’ and final year students’ perspective. In addition, it is designed to report associations and trends between personal values and overall emotional states.

### 1.2 Objectives

1) To identify the common values among medical students.
2) To determine whether there are differences in values in the first and final year of medical education.
3) To determine whether there is a correlation between personal values and emotional state.
4) To determine whether there are any trends of values that align with one’s emotional state.

## 2 Materials and methods

### 2.1 Study design, setting, and participants

This study is an observational analytical cross-sectional study involving first and final year medical students enrolled at the Mohammed Bin Rashid University of Medicine and Health Sciences (MBRU) in the academic year 2023/ 2024. MBRU is part of Dubai Academic Health Corporation, the first integrated academic health system in Dubai, UAE (Dubai Health, 2024). This study was carried out in Dubai with data collection running from December 2023 to January 2024 and data analysis completed in February 2024. Adherence to the STROBE guidelines was maintained in the reporting of this study (Vandenbroucke et al., 2007).

Inclusion criteria for this study encompassed first and final year students enrolled in the college of medicine at MBRU in the academic year 2023/ 2024, and no exclusion criteria were applied during the recruitment process.

### 2.2 Data sources/measurement

The data collection tool consisted of a Google Forms survey sent out to all MBRU students from the college of medicine by email. The survey collected demographic details, followed by two standardized questionnaires assessing exposure and outcome data.

The first questionnaire was the Life Values Inventory (LVI), which contains 42 items that measure 14 relatively independent values (Crace and Brown, 1996). The values measured by the LVI are Achievement, Belonging, Concern for the Environment, Concern for Others, Creativity, Financial Prosperity, Health and Activity, Humility, Independence, Loyalty to Family or Group, Privacy, Scientific Understanding, Responsibility, and Spirituality. The participants were expected to choose the response between (1–5) that best describes “how often the belief guides your behavior”: 1–2 “Seldom guides my behavior,” 3 “Sometimes guides my behavior,” 4–5 “Frequently guides my behavior.”

To assess emotional state, the survey included the Positive and Negative Affect Schedule (PANAS), which evaluates different feelings and emotions (Watson et al., 1988). The participants were expected to choose one of the following five options for each variable: “Very little or not at all,” “A little,” “Moderately,” “Quite a bit,” or “Extremely.” The positive feelings and emotions were interested, excited, enthusiastic, strong, proud, inspired, determined, alert, attentive, and active. The negative feelings and emotions were distressed, upset, guilty, ashamed, scared, afraid, nervous, hostile, irritable, and jittery.

The consent form and survey are available on: https://docs.google.com/document/d/14OuNzAnukdXYkFFOJ8u_BBAOgSuRzToF/edit?usp=sharing&ouid=102743910591107509710&rtpof=true&sd=true

### 2.3 Variables

This study identifies common values, change in individual values and correlations to emotional state while analyzing differences in outcome data by age, gender, and year of study (demographics).

The primary quantitative variable measure is the age of the students.

Each of the PANAS and the LVI component score represented an individual categorical variable ranging from 1 to 5. Each life value in the LVI pertains to the combined score of three of the 42 assessed variables. The LVI scores range from 3 to 15, with higher scores indicating greater importance of the value in guiding behavior. The PANAS scores range from 10 to 50, with higher scores indicating greater intensity of positive or negative emotions.

In the LVI, the value of Achievement encompasses the following variables: Challenging oneself to achieve; Improving performance; Working hard to do better. The value of Belonging comprises: Being liked by others; Being accepted by others; Feeling as though one belongs. Concern for the environment involves: Protecting the environment; Taking care of the environment; Appreciating the beauty of nature. Concern for others encompasses: Being sensitive to others’ needs; Helping others; Being concerned about the rights of others. Creativity involves: Coming up with new ideas; Creating new things or ideas; Discovering new things or ideas. Financial prosperity includes: Having financial success; Making money; Being wealthy. Health and activity entail: Taking care of one’s body; Being in good physical shape; Being strong or good in a sport. Humility is reflected in: Downplaying compliments or praise; Being quiet about one’s success; Avoiding credit for accomplishments. Independence is demonstrated through: Being independent; Giving one’s opinion; Having control over one’s time. Loyalty to family or group is shown by: Accepting one’s place in the family or group; Respecting the traditions of the family or group; Making decisions with the family or group in mind. Privacy involves: Having time to oneself; Having quiet time to think; Having a private place to go. Responsibility encompasses: Being reliable; Being trustworthy; Meeting obligations. Scientific understanding includes: Using science for progress; Knowing things about science; Knowing about math. Spirituality entails: Believing in a higher power; Believing that there is something greater than ourselves; Living in harmony with one’s spiritual beliefs.

The PANAS on the other hand consists of 20 variables which are categorized into positive and negative feelings and emotions. The emotion indicates the emotional state of participants based on how they generally feel on average with no time-period specified. As there are 10 positive and 10 negative emotions accounted for, each participant’s scores ranged from a minimum of 10 to a maximum of 50 for each set of positive or negative emotions.

The demographic data included gender, male or female; year of expected graduation 2024 or 2029; and age.

### 2.4 Study size

All MBRU students in first (*n* = 82) and last (*n* = 45) academic years were eligible to participate in the study. Thus, no sample size was statistically calculated as it was based on obtaining complete coverage. The final sample size was 84 students.

### 2.5 Statistical methods

In this study, statistical analysis was conducted using the Statistical Package for Social Sciences (SPSS), version 28 software. A significance level of *p* ≤ 0.05 was employed to determine statistical significance. To assess the strength and direction of relationships between continuous variables, Pearson’s correlation coefficient (r) was utilized. The choice of the independent samples t-test for comparing means of two independent groups was based on its suitability for detecting differences between groups. Individual scores for emotions in PANAS and values in LVI were calculated using the sum function in Microsoft Excel. The normality assumption was tested using Kolmogorov–Smirnov test separately for Positive and Negative Affect Schedule. The instrument reliability as measured by Cronbach’s alpha is 0.945 (95% CI: 0.927, 0.961).

## 3 Results

**Figure 1:**
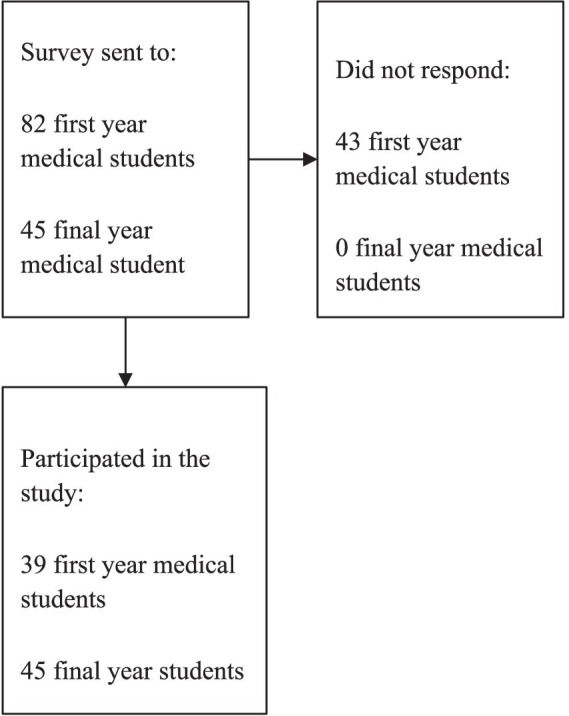
Flow chart of participants.

The survey was distributed to all first-year and final-year medical students. Out of 129 eligible students, 84 participated (response rate of 65.1%). The final sample included 39 (out of 82) first-year students and 45 (out of 45) final-year students (Figure 1).

Slightly over half were final-year students (45/84; 53.6%), and the majority were females (70/84; 83.3%). The eligible sample consisted of 19 first-year males and 63 first-year females (total 82). For final-year students, there were 6 males and 39 females (total 45). The majority of medical students were female, resulting in a gender imbalance.

First-year students ranged in age from 17 to 23 years, while final-year students ranged from 22 to 33 years. The mean (SD) age in first year medical students was 18.33 (1.3). The mean (SD) age in final year medical students was 23.37 (2.0). No additional demographic data were collected to ensure confidentiality.

The highest mean score (SD) for life values was Privacy, 13 (2) out of 15. This was followed by the life values of Spirituality, Scientific Understanding, Responsibility, Independence and Belonging, with a mean (SD) of 12 (2) out of 15. While the mean (SD) of the life value Achievement was 11(3). For the values Concern for the Environment, Creativity, Financial Prosperity, Health and Activity, Humility, Loyalty to Family or Group the mean (SD) was 11 (2). The lowest mean score of 10 (2) was for the life value of Concern for Others.

**Figure 2:**
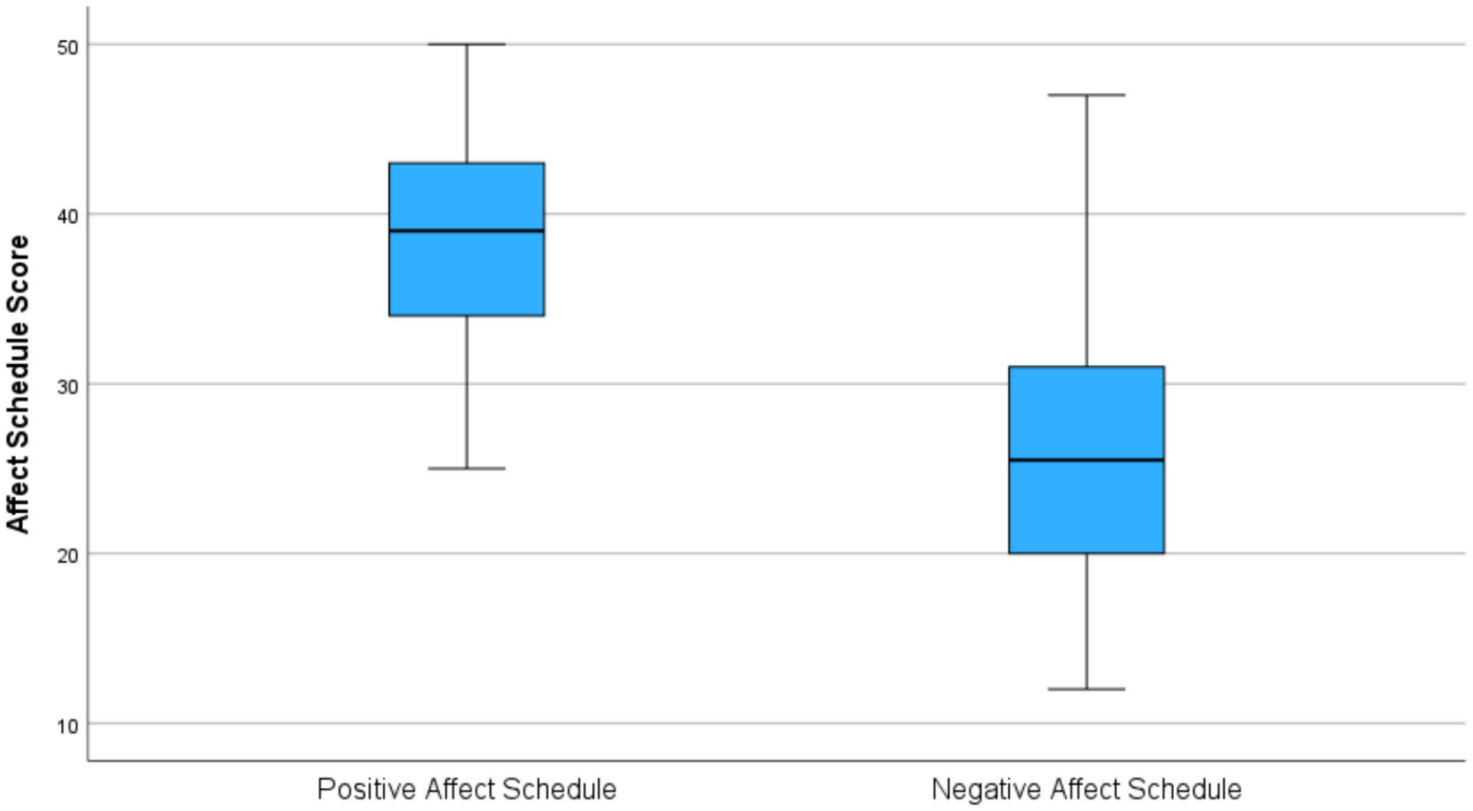
The distribution of the Positive and Negative Affect Schedule (PANAS).

Regarding the findings of the PANAS, the mean score (SD) of positive emotions was 38 (6) out of 50. The mean score of negative emotions was 26 (7) out of 50 (Figure 2).

Table 1 presents correlations between the scores of LVI with the scores of PANAS. There is a significant correlation between positive emotions and the life values of Achievement, Creativity, Concern for Environment, Financial Prosperity, Health and Activity, Privacy, Responsibility, Scientific Understanding, and Belonging (*p* < 0.05). In addition, there is a significant correlation between negative emotions and the life values of Achievement, Health and Activity, Scientific Understanding, and Spirituality (*p* < 0.05).

**Table 1:** Statistically significant correlations between the Life Values Inventory (LVI) and the Positive and Negative Affect Schedule (PANAS).

LVI	PANAS	Correlation	Count	Lower C.I.	Upper C.I.	*p* value
Achievement	Positive emotions	0.38	84	0.18	0.55	*p* < 0.05
	Negative emotions	−0.31	84	−0.49	−0.11	*p* < 0.05
Creativity	Positive emotions	0.29	84	0.08	0.47	*p* < 0.05
Concern for the environment	Positive emotions	0.23	84	0.02	0.43	*p* < 0.05
Financial prosperity	Positive emotions	0.21	84	0.00	0.41	*p* < 0.05
Health and activity	Positive emotions	0.34	84	0.13	0.51	*p* < 0.05
	Negative emotions	−0.23	84	−0.42	−0.02	*p* < 0.05
Privacy	Positive emotions	0.23	84	0.02	0.43	*p* < 0.05
Responsibility	Positive emotions	0.23	84	0.02	0.42	*p* < 0.05
Scientific understanding	Positive emotions	0.27	84	0.06	0.45	*p* < 0.05
	Negative emotions	−0.28	84	−0.47	−0.07	*p* < 0.05
Spirituality	Negative emotions	−0.26	84	−0.45	−0.05	*p* < 0.05
Belonging	Positive emotions	0.22	84	0.00	0.41	*p* < 0.05

LVI, Life Values Inventory; PANAS, Positive and Negative Affect Schedule; C.I., Confidence Interval.

Table 2 displays correlations between gender and the scores of LVI and PANAS. Life values guided by humility and independence were significantly associated with gender. The life value of Humility was significantly associated with gender (*p* = 0.031) with a mean score (SD) of 11 (2) out of 15 in females and mean score (SD) of 10 (3) out of 15 in males. The life value of Independence was also significantly associated with gender (*p* = 0.048) with a mean (SD) of 12 (2) out of 15 in females and 11(2) out of 15 in males. There was a possible association between the life value of Concern for the Environment and gender (*p* = 0.051) with a mean (SD) of 11 (2) in females and a mean (SD) of 12 (2) in males.

**Table 2:** Mean and standard deviation of scores of the Life Values Inventory (LVI) and the Positive and Negative Affect Schedule (PANAS) according to gender.

	Female	Male	*p* value
	Mean (SD)	Mean (SD)	
Achievement	11.33 (2.58)	11.79 (2.29)	0.270
Belonging	11.69 (2.53)	11.93 (2.13)	0.369
Concern for the environment	11.14 (2.38)	12.29 (2.30)	0.051
Concern for others	10.44 (1.95)	10.64 (2.47)	0.370
Creativity	10.80 (2.19)	11.14 (2.32)	0.299
Financial prosperity	11.00 (2.06)	11.50 (1.91)	0.203
Health and activity	10.83 (2.28)	11.36 (2.27)	0.215
Humility	10.81 (2.13)	9.57 (2.74)	0.031*
Independence	12.14 (2.26)	11.07 (1.69)	0.048*
Loyalty to family or group	11.21 (2.04)	11.57 (1.91)	0.274
Privacy	12.76 (2.31)	12.93 (2.06)	0.398
Responsibility	12.27 (2.33)	11.86 (2.32)	0.272
Scientific understanding	11.81 (2.40)	11.93 (2.02)	0.434
Spirituality	11.57 (2.01)	11.21 (2.83)	0.287
Positive emotions (PANAS)	37.29 (6.25)	41.50 (4.15)	0.009*
Negative emotions (PANAS)	26.71 (6.86)	21.29 (8.98)	0.006*

*, statistically significant; SD, Standard Deviation.

For PANAS, positive emotions were significantly associated with gender (*p* = 0.009) with a mean score (SD) of 37 (6) out of 50 in females and 42 (4) out of 50 in males. Negative emotions were significantly associated with gender (*p* = 0.006) with a mean score (SD) of 27 (7) out of 50 in females and 21 (9) out of 50 in males.

Table 3 shows correlations between year of study and LVI mean scores. Privacy had the highest mean score (SD) of 13 (2) out of 15 in both final year and first year students (*p* = 0.21). The life values of Independence (*p* = 0.06), Responsibility (*p* = 0.46), and Scientific Understanding (*p* = 0.44) had a mean score (SD) of 12 (2) out 15 in both final year and first year medical students. Achievement was significantly correlated (*p* = 0.04) with the year of study with a mean (SD) of 12 (2) out of 15 in first year students and a mean (SD) of 11 (3) out of 15 in final year students.

**Table 3:** Mean and standard deviation of scores of the Life Values Inventory (LVI) by year of study.

Life values inventory	Year 1	Year 6	*p* value
	Mean (SD)	Mean (SD)	
Achievement	11.92 (2.18)	10.96 (2.74)	0.040*
Belonging	12.08 (2.31)	11.42 (2.56)	0.113
Concern for the environment	11.46 (2.10)	11.22 (2.64)	0.320
Concern for others	10.72 (1.95)	10.27 (2.10)	0.157
Creativity	11.03 (2.25)	10.71 (2.17)	0.259
Financial prosperity	11.21 (2.02)	10.98 (2.07)	0.306
Health and activity	11.05 (2.16)	10.80 (2.38)	0.308
Humility	10.74 (2.30)	10.49 (2.26)	0.306
Independence	12.36 (2.03)	11.62 (2.31)	0.064
Loyalty to family or group	11.26 (1.82)	11.29 (2.19)	0.471
Privacy	13.00 (2.08)	12.60 (2.41)	0.210
Responsibility	12.23 (2.32)	12.18 (2.34)	0.459
Scientific understanding	11.87 (2.18)	11.80 (2.47)	0.444
Spirituality	11.72 (2.13)	11.33 (2.18)	0.229

*, statistically significant; SD, Standard Deviation.

Table 4 shows correlations between year of study and PANAS scores. Positive emotions on average were scored high in both academic years with mean (SD) of 40 (5) out of 50 in year 1 and mean (SD) of 36 (7) out of 50 in year 6 (*p* = 0.006). Negative emotions were on average scored lower than positive emotions with a mean (SD) of 25 (8) out 50 in year 1 and 26 (7) out 50 in year 6 (*p* = 0.46).

**Table 4:** Correlation between year of study and scores of the Positive and Negative Affect Schedule.

	Year 1	Year 6	Correlation coefficient	*p* value
	Mean (SD)	Mean (SD)		
Positive emotions	40 (5)	36 (7)	0.27	0.006*
Negative emotions	25 (5)	26 (7)	−0.08	0.458

*, statistically significant; SD, Standard Deviation.

## 4 Discussion

Values play a pivotal role in shaping the identities and decision-making processes of medical students, reflecting their intrinsic motivations and guiding their professional development (Tak et al., 2017; Ratanawongsa et al., 2006). Our study sheds light on the evolving landscape of medical education, emphasizing the importance of cultivating values that promote empathy, integrity, and resilience among future healthcare professionals (Gogo et al., 2019; Epstein and Krasner, 2013).

Trends in medical student values reveal changes in perceptions and priorities. Chin (2002) emphasized the significance of humility and empowerment in fostering a humanistic approach to medical practice. Our findings support this, showing prevalent values such as responsibility, humility, and concern for others across different academic years.

The correlation between personal values and emotional state shows the interconnectedness of psychological factors with professional development. Previous studies have suggested that nurturing empathy and compassion can enhance patient care outcomes (Wear and Zarconi, 2015; Patel et al., 2019).

Our study extends the perspective by demonstrating significant correlations between positive emotions and values related to achievement, belonging, and creativity. These findings suggest that fostering these values may enhance resilience among medical students. Resilience, the ability to adapt and thrive despite challenges (Rose and Palattiyil, 2020), is crucial in medical education. By promoting values that align with positive emotions, educators can help students build resilience, improving their well-being and professional development.

Gender differences in values and emotional state further highlight the nuanced interplay between individual characteristics and professional identity formation. Samuriwo et al. (2020) emphasized the importance of reflective practice in fostering professional growth, suggesting that gender-related differences in values may influence students’ perceptions of their roles and responsibilities in healthcare settings. Our findings reveal significant associations between gender and values such as humility and independence.

Academic progression impacts values and emotions, underscoring the dynamic nature of medical education. Dyrbye et al. (2008) highlighted burnout prevalence among medical students, emphasizing the need for proactive interventions to support students’ mental health. Our study shows that while positive emotions remain high across academic years, they decrease over time. Negative emotions remain stable but consistently lower, indicating a persistent aspect of emotional experiences. These findings underscore the importance of ongoing support initiatives and curriculum development efforts tailored to address the evolving emotional needs of medical students as they progress through their educational journey.

### 4.1 Implications for public health educators

Our findings hold several implications for public health educators involved in medical education. Firstly, the correlation between personal values and emotions underscores the importance of integrating values-based education into the curriculum. By incorporating reflective exercises and discussions on values alignment with professional roles, educators can foster a sense of purpose and resilience among medical students (Holden et al., 2015; Wald et al., 2015). Additionally, understanding the gender differences in values and emotions can inform targeted interventions aimed at addressing specific needs and challenges faced by male and female students.

Based on our findings, support initiatives should include targeted mental health interventions and values-based training programs to enhance resilience and emotional well-being among medical students. These programs could involve reflective exercises, mentorship, promoting collaborative work, and workshops focused on aligning personal values with professional roles.

### 4.2 Strengths, limitations, & generalizability

Strengths of our study include targeting students at the beginning and at the end of their medical school education, which allowed for the comprehensive assessment of values and emotional states among medical students at different stages of their academic journey. Moreover, the use of standardized questionnaires facilitated the collection of reliable data, enhancing the robustness of our findings. However, several limitations should be acknowledged. Firstly, the study was conducted at a single institution, limiting the generalizability of our findings to other medical schools or cultural contexts. One limitation of our study is the different sample sizes of first-year and final-year students, which may affect the comparability of mean values. The non-response from 43 first-year medical students could be attributed to lack of interest or being busy with other academic requirements. Future studies should consider balanced sample sizes to ensure more robust comparisons. Additionally, the self-reported nature of the data may be subject to social desirability bias, potentially influencing participants’ responses. However, the use of anonymous surveys in our study has likely minimized this risk. Future studies could address these limitations by employing longitudinal designs and incorporating qualitative methods to gain deeper insights into students’ experiences and perspectives.

### 4.3 Areas for future research

While our study provides valuable insights into the relationship between values and emotional states among medical students, several avenues for future research warrant exploration. Firstly, longitudinal studies could elucidate the trajectory of values development throughout medical education, as well as healthcare students in general, and its impact on professional identity formation. Additionally, qualitative inquiries could provide rich insights into the underlying mechanisms driving the observed correlations between values and emotional states. Furthermore, interventions aimed at promoting values-aligned well-being among healthcare students could be evaluated for their effectiveness in enhancing resilience and mitigating burnout. By addressing these gaps in the literature, future research can contribute to the ongoing efforts to optimize medical education and support the flourishing of future generations of healthcare professionals.

## 5 Conclusion

Our study adds to the literature on values and emotional well-being in medical education by demonstrating significant correlations between personal values and emotional states among medical students. It emphasizes the need to integrate values-based education into medical curricula to promote empathy, integrity, and resilience, which are vital for the professional growth of future healthcare providers.

Understanding the intricate relationship between values and emotions is essential for educators and policymakers to support the comprehensive development of future healthcare professionals. By fostering this alignment, we can cultivate a more resilient and fulfilled healthcare workforce ready to navigate the challenges of their profession.

## Data Availability

The original contributions presented in the study are included in the article/supplementary material, further inquiries can be directed to the corresponding author.
